# Bivariate association analysis of longitudinal phenotypes in families

**DOI:** 10.1186/1753-6561-8-S1-S90

**Published:** 2014-06-17

**Authors:** Phillip E Melton, Laura A Almasy

**Affiliations:** 1 Centre for Genetic Origins of Health and Disease, University of Western Australia, Crawley, Australia; 2 Department of Genetics, Texas Biomedical Research Institute, San Antonio, USA

## Abstract

Statistical genetic methods incorporating temporal variation allow for greater understanding of genetic architecture and consistency of biological variation influencing development of complex diseases. This study proposes a bivariate association method jointly testing association of two quantitative phenotypic measures from different time points. Measured genotype association was analyzed for single-nucleotide polymorphisms (SNPs) for systolic blood pressure (SBP) from the first and third visits using 200 simulated Genetic Analysis Workshop 18 (GAW18) replicates. Bivariate association, in which the effect of an SNP on the mean trait values of the two phenotypes is constrained to be equal for both measures and is included as a covariate in the analysis, was compared with a bivariate analysis in which the effect of an SNP was estimated separately for the two measures and univariate association analyses in 9 SNPs that explained greater than 0.001% SBP variance over all 200 GAW18 replicates.The SNP 3_48040283 was significantly associated with SBP in all 200 replicates with the constrained bivariate method providing increased signal over the unconstrained bivariate method. This method improved signal in all 9 SNPs with simulated effects on SBP for nominal significance (*p-*value <0.05). However, this appears to be determined by the effect size of the SNP on the phenotype. This bivariate association method applied to longitudinal data improves genetic signal for quantitative traits when the effect size of the variant is moderate to large.

## Background

Traditional analyses of genetic variants influencing complex diseases focus on phenotypes and covariate measurements from a single time point. However, the majority of human epidemiologic studies collect information from multiple measurements. This, coupled with the knowledge that many quantitative phenotypes correlated with complex disease change with age or environmental confounders, suggests that inclusion of a temporal component may allow for increased understanding of complex diseases. Given the nature of these longitudinal data, methods jointly using multiple time points when performing association may have increased statistical power over univariate association methods [1-6]. However, although some statistical methods have been proposed for the analysis of longitudinal data, few have been successful in being adopted by the wider genetic epidemiologic community because of the difficulty of implementing them. One potential drawback to the utility of these bivariate methods is the addition of a degree of freedom as a result of the additional phenotype, thereby potentially reducing statistical power to detect genetic signals that do not vary with time or age.

We present a method for bivariate association using longitudinal data from the same phenotype in families using the Genetic Analysis Workshop 18 (GAW18) simulated single-nucleotide polymorphism (SNP) data for the phenotype systolic blood pressure (SBP) from visits 1 and 3. We have previously applied this method to the analysis of different phenotypic measures of heart rate (echo- and electrocardiograms) in American Indian participants of the Strong Heart Family Study [7] but wish to test its efficacy in a simulated longitudinal data set. To test this method, we first conducted association using measured genotype analysis of all SNPs for SBP from visits 1 and 3 using the GAW18 family data. We then conducted two bivariate analyses within the variance-component framework using 20 SNPs known to influence SBP from the GAW18 SNPs and 20 SNPs that did not explain any of the SBP variance identified in our association analysis. This work was done with knowledge of the GAW18 simulating model.

## Methods

### Data description

The GAW18 data set contains 959 individuals from 20 extended Mexican American pedigrees from the Type 2 Diabetes Consortium. Each of the 200 simulated data sets includes the following information for each individual for three time periods along with gender: age, SBP, diastolic blood pressure (DBP), hypertension status, blood pressure medication status, and smoking [8].

### Univariate association

Maximum likelihood methods, taking into account relationships among family members, were used to determine association for the phenotypes SBP at visit 1 (SBP_1) and visit 3 (SBP_3) independently in a polygenic model available in the computer program Sequential Oligogenic Linkage Analysis Routines (SOLAR) [9]. Covariates included age, sex, and their interactions as well as smoking for both visits 1 and 3. Variables were carried forward to association models if associated with SBP_1 or SBP_3 at *p-*valuebelow0.05. Measured genotype analysis was conducted for all available GAW18 polymorphic variants in which the number of minor alleles is added to the quantitative polygenic genetic model as a covariate to assess the effect of the SNP on the mean of the trait using the equation

(1) p=μ+αs+βx+g+e,

where *s *defines a variate for the *i*th SNP that takes the value, 0, 1, and 2 for the marker genotypes AA, Aa, and aa, respectively; α represents one-half the displacement between homozygous marker means; β represents fixed-effect regression coefficients for any measured covariates *x*; and *g *and *e *are random effects representing residual genetic effects and random environmental effects [10]. This model tests whether *α *is different from 0 using a likelihood ratio test. Twice the difference in log-likelihoods is distributed as a $x^{2}$ random variable with 1 degree of freedom.

### Bivariate association

We also applied maximum likelihood methods accounting for familial relationships in bivariate association analyses. This bivariate method investigates two related phenotypes simultaneously, modeling genetic and environmental correlations between them [11]. Our proposed method investigates the effect of an SNP on the mean trait values of two longitudinal phenotypes *i *and *j*, constraining the displacement in trait means (α) with each copy of the minor allele to be equal for both measures using the equations

(2) $${\text{p}}_{\text{i}}={\text{μ}}_{\text{i}}+{\text{α}}_{\text{i}}{\text{s}}_{\text{i}}+\text{Σ}{\text{β}}_{\text{i}}{\text{x}}_{\text{i}}+{\text{g}}_{\text{i}}+{\text{e}}_{\text{i}}\;\text{and}$$

(3) $${\text{p}}_{\text{j}}={\text{μ}}_{\text{j}}+{\text{α}}_{\text{j}}{\text{s}}_{\text{j}}+\text{Σ}{\text{β}}_{\text{j}}{\text{x}}_{\text{j}}+{\text{g}}_{\text{j}}+{\text{e}}_{\text{j}},$$

where α, β*_i_*, and β*_j _*are fixed-effect regression coefficients and *g *and *e *are modeled through random effects with the bivariate model allowing for correlations between *g_i _*and *g_j _*(ρ*_g_*) and between *e_i _*and *e_j _*(ρ*_e_*). The difference between the log-likelihoods of a model in which the SNP effect is estimated versus one in which it is constrained to zero is then distributed as a $x^{2}$ distribution with 1 degree of freedom.

For our bivariate analysis, we used the same covariates from the univariate analysis along with 9 variants that explained greater than 0.001 of SBP variance from the GAW18 answers.We then compared these results with univariate association models and a bivariate model in which the effect of genotype on the mean trait value of the two phenotypes was estimated separately, distributed as a $x^{2}$ distribution with 2 degrees of freedom.Results were compared between approaches over 200 GAW18 replicates to determine which method provided the best evidence for genetic signal for these SNPs, tallying the proportion of replicates in which association was detected at *p*-values below $0.001,\;5.0\times 10^{-5},$ and $5.0\times 10^{-9}$.

## Results

The average genetic correlation (ρ*_g_*) for SBP over 200 GAW18 replicates between visits 1 and 3 was $0.971\left(\pm 0.029\right)$ with an average environmental correlation of $0.486\left(\pm 0.029\right)$. This high ρ*_g_*value demonstrates that these two phenotypes are measures of the same genetic mechanism and therefore appropriate for our proposed bivariate association approach.

### Univariate association

Table 1 shows results of three different association analyses for 9 SNPs influencing SBP across all 200 GAW18 replicates for *p*-values below 0.05, 0.001, and $5.0\times 10^{-9}$. All analyses identified the variant 3_48040283 in *MAP4 *as genome-wide significant $\left(p-\text{value}<5.0\times 10^{-9}\right)$. The *MAP4 *SNP, 3_47957996 was significant in 199 of the constrained bivariate tests and 200 of the unconstrained tests, with the number of genome-wide significant replicates dropping slightly for univariate models. Two additional variants, 1_66075952 from *LEPR *and *MAP4 *variant 3_28601297, demonstrated low numbers of genome-wide significant associations across the four tested association methods.

**Table 1:** Comparisons of association analyses results for 9 functional variants explaining more than 0.001 of the trait variance.

Variant (%Variance SBP^1^)	Bivariate constrained				Bivariate unconstrained			Univariate visit 1			Univariate visit 3	
	
	0.001	5.0 × 10^−5^	5.0 × 10^−9^	0.001	5.0 × 10^−5^	5.0 × 10^−9^	0.001	5.0 × 10^−5^	5.0 × 10^−9^	0.001	5.0 × 10^−5^	5.0 × 10^−9^
3_48040283 (0.0278)	200^2^	200	200	200	200	200	200	200	199	200	200	197
1_66075952 (0.0206)	153	76	1	121	50	1	137	64	1	125	57	2
3_47957996 (0.0149)	200	200	199	200	200	200	200	200	193	200	200	195
3_47956424 (0.0143)	182	133	3	177	111	4	169	108	5	169	115	3
3_48040284 (0.011)	49	14	0	33	8	0	24	4	0	30	7	0
13_28624294 (0.0081)	26	0	0	4	0	0	11	1	0	18	4	0
3_47913455 (0.004)	11	1	0	8	1	0	3	0	0	5	0	0
3_58109162 (0.0027)	41	9	0	22	5	0	13	0	0	8	0	0
19_12541795 (0.0017)	0	0	0	0	0	0	0	0	0	0	0	0

^1^Percent of the variance explained by the variant for SBP from the Genetic Analysis Workshop 18 (GAW18) answers.

^2^Number of replicates exceeding threshold.

### Bivariate association

When comparing the different methods, the bivariate method in which the effect of genotype on mean trait values of two phenotypes is constrained to be equal provided the most robust analysis, improving association for all 9 variants compared with the bivariate analysis in which these values were estimated separately and versus univariate analyses of exam 1 and 3 in cases where the *p*-value is less than 0.001 or *p*is below 5.0 × 10^−5^. To ensure that the improved power for the constrained bivariate approach did not come at the expense of increased false-positive rates, we chose 20 SNPs that did not explain any of the variance from the simulated model. For these 20 null markers, there were an average of 8.1 replicates less than 0.05 for the constrained bivariate (range, 1-28), indicating no systematic inflation of *p*-values under the null (data not shown).

## Discussion

The analysis of genetic variants using longitudinal data has the potential to be a valuable resource for determining biological and environmental factors affecting complex disease phenotypes over time. This type of analysis may provide increased power to detect rare genetic variants in complex diseases or to better understand when genetic components contribute to human development [4]. In addition, these types of analyses may allow for the identification of environmental covariates associated with complex diseases[2]. However, although statistical genetic methods for the analysis of longitudinal data have been proposed, they have not been widely adopted. The single degree of freedom association test we propose could also be implemented easily in generalized estimating equations (GEEs) or other mixed-model frameworks. However, theoretical advantages to using the likelihood-based variance component framework are that the bivariate variance component model explicitly allows both shared/stable and unshared/changing genetic and environmental effects across timeand age in the random effects portion of the model through the estimation of genetic and environmental correlations.

## Conclusions

In this paper, we present a bivariate approach to increase the genetic signal for a variant by constraining the effect of the SNP on the phenotype using a variance-component model. This model is predicated on the assumption that there is no gene- by-age interaction; however, the structure is general and is applicable to other issues in genetic epidemiology. As whole-genome data becomes more affordable for large-scale epidemiologic studies, an important consideration will be to maximize the ability to detect rare variants that have a large effect on complex disease. The easiest way to detect these rare variants will be through large pedigrees because they are amplified in families. However, the sample size of family studies is often small, making it difficult to determine association; therefore, methodologies that maximize the use of the genetic data and phenotypes from longitudinal studies may allow for an increased ability to identify genetic variants associated with complex disease. The model presented in this manuscript can be used as an early step in the analysis of longitudinal data and may lead to the development of more complex models.

## Competing interests

There are no competing interests.

## Authors' contributions

LA designed the overall study. PM conducted the analysis and drafted the manuscript. All authors read and accepted the final manuscript.
